# *The Plant Genome* special section: Epigenome and epitranscriptome in plant–environment interactions

**DOI:** 10.1002/tpg2.20404

**Published:** 2023-12-20

**Authors:** Wai‐Shing Yung, Ting‐Fung Chan, Fanjiang Kong, Hon‐Ming Lam

**Affiliations:** ^1^ School of Life Sciences and Center for Soybean Research of the State Key Laboratory of Agrobiotechnology The Chinese University of Hong Kong Hong Kong SAR P. R. China; ^2^ Innovative Center of Molecular Genetics and Evolution, School of Life Sciences Guangzhou University Guangzhou P. R. China; ^3^ Institute of Environment, Energy, and Sustainability The Chinese University of Hong Kong Hong Kong SAR P. R. China

Understanding how plants interact with the external environment is critical for improving crops' adaptation to environmental stresses and restoring marginal lands to fulfill food demand under the changing climate. In response to environmental cues, plants utilize the dynamic epigenome to modulate and fine‐tune the temporal gene expression for development and stress adaptation (Lloyd & Lister, 2022). During long‐term adaptation, certain epigenetic modifications can even be retained during aging and transmitted to the progenies (Lamke & Baurle, 2017). Over the past decades, technological advances have allowed researchers to have enriched knowledge about the dynamic changes in epigenetic modifications at the genomic level in different plant species (Perrone & Martinelli, 2020). Considering the emerging number of epigenomic studies in plants, there is a need to effectively integrate the epigenomic information obtained to generate a holistic understanding of epigenetic regulations in plant responses to the external environment. In moving forward to the investigation of crops, revisiting the concepts and focuses of epigenetic studies would pave the way to potential applications to tackle the challenges posed by the changing environment on crop production. Besides, the epitranscriptome featured by numerous types of RNA modifications is also an important regulatory layer of gene expression (Meyer & Jaffrey, 2014). The revolutionary development of third‐generation sequencing technologies has enabled comprehensive analyses of plant epitranscriptome and greatly benefited the deciphering of gene regulation to provide novel mechanistic insights and strategies for crop improvement (Zhao et al., 2019). This special issue has collected eight articles surrounding the described themes and the highlights are summarized here.

Flowering is a developmental process well known to be regulated by epigenetic mechanisms in plants (He et al., 2003). The accurate perception of external environmental conditions enables flowering at the right time. As one of the major pathways controlling flowering time, the photoperiod pathway integrates the signal of day length into the regulation of *CONSTANS* (*CO*) and thus *FLOWERING LOCUS T* (*FT*) expression which induces flowering. Liu et al. (2023) reviewed the current knowledge on the histone‐modifying enzymes responsible for altering the chromatin statuses of the important regulatory genes of photoperiodic flowering, including *FT*, *CO*, and GIGANTEA, and reiterated the lack of evidence showing concrete relationships between photoperiodic control and other epigenetic features such as DNA methylation and chromatin remodeling in *Arabidopsis*. While rice displays photoperiodic regulation similar to *Arabidopsis*, the pathway in soybean involves regulatory components specific to legumes, suggesting the promising role of epigenetic regulation to be delineated for crop improvement.

Meanwhile, an increasing number of reports have profiled the epigenetic features of legume crops in response to abiotic stresses and they are reviewed by Yung, Huang et al. (2022). While past attempts focused on the changes in the total level of DNA methylation and histone modification, technological advances have facilitated recent studies to define extensive numbers of differential regions in the genome of several legume species such as soybean, castor bean, and mungbean. In addition to the efforts made on genome‐wide identification, functional characterization of microRNAs was performed to reveal their roles in inhibiting key regulators in stress response and tolerance. Future studies need to integrate the information from multiple layers to reconstruct the chromatin status for a better interpretation of the regulatory consequences of epigenetic changes during different stress episodes and across generations.

Fresnedo‐Ramirez et al. (2023) revisited the theoretical framework of epigenetic mechanisms in model plants and emphasized the importance of extending the current knowledge to crop species. Using the recent findings in almond as examples, the complexity of epigenetic regulation in perennial crop plants was demonstrated. DNA methylation was not only associated with bud dormancy and flowering time as encountered in model plant species but also found to be the underlying factor of self‐incompatibility and noninfectious bud failure (NBF) in almond. As heat stress and aging are associated with the severity of NBF, this disorder would greatly hamper almond production in the changing climate. The findings on differential DNA methylations associated with gene expression under NBF shed light on the utilization of epigenetic changes as an alternative approach to tackle challenges in crop production imposed by climate change.

Long noncoding RNAs (lncRNAs) comprise a group of nonprotein coding transcripts that have diversified modes of action and functions in plants. Yuan et al. (2022) summarized the role of lncRNAs in influencing the synthesis and action of small RNAs. When plants are exposed to temperature stresses, lncRNAs participate in inhibiting the expression of key transcription factors after their initial upregulation to restore the normal growth status. While it was well evidenced that lncRNAs acted as regulators of salt stress tolerance and drought tolerance, the mechanistic basis of their induced expression has been less explored. It should be noticed that the lack of conserved function of lncRNAs raises the need for the identification and characterization of important members in different plant species during their interaction with diversified environmental stimuli. Recent research scope about noncoding RNAs (ncRNAs) has also been extended into their functions in the interaction between plants and other organisms (Song et al., 2021). Increasing evidence has shown that noncoding RNAs could be exported from one organism and perform biological functions in the interacting organism. As reviewed by T. Liu, Xu et al. (2022), such bi‐directional ncRNA transport was reported in plant pathogenesis in which ncRNAs were used by each party to silence immunity or virulence genes in the other party. Similarly, the artificial expression of dsRNA in plants can eventually lead to small RNA production and gene silencing in herbivorous insects, suggesting potential applications in pest control. On the other hand, ncRNA transport across organisms may also have beneficial effects, as shown in the case of promoting nodule formation during legume‐rhizobium symbiosis. While phenotypic effects can be observed, the processes and machineries responsible for the cross‐kingdom transport and uptake remain elusive. The summarized insights on the factors controlling ncRNA export and transport, such as the identity of specific RNA‐binding proteins, chemical modifications of ncRNA, and the composition of extracellular vesicles, are fundamental in understanding their regulatory mechanisms and benefiting potential applications.

Adapting to climate change is also essential to forest trees, in which variation of gene expression plays a vital role in creating phenotypic plasticity (M. Liu, Liu et al., 2022). To elucidate the effects of genetic factors and environmental factors on the gene expression of Siberian larch (*Larix sibirica* Ledeb.), transcriptomic annotation and expression analysis of the trees grown in the natural environment were performed. It was reported that the differential gene expression between the trees along the mountain was mainly contributed by the variations in altitude between plots rather than the genetic variations. Co‐expression analysis further illustrated that different modules, including those featuring genes related to heat stress and basal metabolism, were correlated with the differences in temperature and altitude. Given the importance of varying gene expression in the adaptation of Siberian larch to different environmental conditions, the potential contribution of epigenetic effects in this perennial tree species awaits further studies.

Transcription factors are key players in plant stress response by regulating the expression of effectors for stress adaptation. While the chromatin of transcription factor genes is frequently marked by different epigenetic modifications, recent research reported the participation of transcription factors in the protein complexes responsible for epigenetic regulation (Luo et al., 2023; Yung, Wang et al., 2022). Chen et al. (2022) identified WD40 transcription factor genes in walnut tree using transcriptomic data and explored their responses to temperature stress. The expression pattern of 42 WD40 genes was investigated at different time points under high‐temperature and low‐temperature stress, leading to the identification of gene candidates for future characterization. Considering the evidence that WD40‐repeat protein family members can play a central role in mediating histone modifications in *Arabidopsis* and wheat (Jiang et al., 2011; Liu et al., 2019), this study shed light on the exploration of the related mechanism in woody plants.

In addition to the components in transcriptional regulation, the epitranscriptome represents another regulatory layer of gene expression (Wiener & Schwartz, 2021). The epitranscriptome comprises the dynamic profile of RNA transcripts that are chemically modified co‐transcriptionally or post‐transcriptionally. In combination with RNA processing events such as alternative splicing, the modification and organization of RNA transcript influence the transcriptional output that determines developmental fate and responses to the external environment. Xie et al. (2023) presented the advances in the technical platforms for epitranscriptomic profiling and explained the technical concerns should be addressed in the workflow of third‐generation sequencing methods, including PacBio SMRT sequencing and Oxford Nanopore Technologies direct RNA sequencing. The power of long‐read sequencing in preserving the information about different modifications in the same RNA molecule greatly benefits the interpretation of their role in regulating RNA stability and processing. While several reports indicated the involvement of RNA modification and editing in abiotic stress responses in the model plant, the application of third‐generation sequencing in the investigation of the epitranscriptome in crop species is promising.

## AUTHOR CONTRIBUTIONS

**Wai‐Shing Yung**: Writing—original draft; writing—review and editing. **Ting‐Fung Chan**: Writing—review and editing. **Fanjiang Kong**: Writing—review and editing. **Hon‐Ming Lam**: Writing—original draft; writing—review and editing.

## CONFLICT OF INTEREST STATEMENT

The authors declare no conflicts of interest.
